# Web-Based Physical Activity Intervention for Latina Adolescents: Feasibility, Acceptability, and Potential Efficacy of the Niñas Saludables Study

**DOI:** 10.2196/jmir.9206

**Published:** 2018-05-09

**Authors:** Britta Larsen, Tanya Benitez, Mayra Cano, Shira S Dunsiger, Bess H Marcus, Andrea Mendoza-Vasconez, James F Sallis, Michelle Zive

**Affiliations:** ^1^ Department of Family Medicine & Public Health University of California, San Diego San Diego, CA United States; ^2^ Centers for Behavioral and Preventive Medicine Department of Psychiatry and Human Behavior Miriam Hospital Providence, RI United States; ^3^ Department of Behavioral and Social Sciences Brown University School of Public Health Providence, RI United States; ^4^ Center for Community Health Department of Pediatrics University of California, San Diego San Diego, CA United States

**Keywords:** exercise, health behavior, internet, eHealth, telemedicine

## Abstract

**Background:**

Physical activity is markedly low in Latina adolescents, yet few physical activity interventions have been attempted in this population. Web-based interventions can incorporate theory-based components, be appealing to adolescents, and have potential for low-cost dissemination.

**Objective:**

This study aimed to assess the feasibility, acceptability, and potential efficacy of a Web-based physical activity intervention for Latina adolescents in a single-arm pilot trial.

**Methods:**

A total of 21 Latina adolescents (aged 12-18 years) who could read and write in English and were underactive (<90 min/week) participated in a 12-week, theory-informed Web-based physical activity intervention. The intervention website was modified from a previous Web-based intervention for Latina adults. Web content was individually tailored based on the responses to monthly questionnaires. Feasibility was measured by recruitment, retention, and adherence/engagement, and acceptability was measured by satisfaction surveys. Physical activity was measured at baseline and follow-up (12 weeks) using the 7-day physical activity recall (PAR) interview and accelerometers.

**Results:**

Baseline activity as measured by the 7-day PAR and accelerometers was 24.7 (SD 26.11) and 24.8 (SD 38.3) min/week, respectively. At 12 weeks, 19 participants (90%, 19/21) returned. Adherence and engagement with materials were low, but 72% (15/21) of the participants indicated that they were satisfied with the intervention. Activity at 12 weeks increased by 58.8 (SD 11.33) min/week measured by the 7-day PAR (*P*<.001). Accelerometer-measured activity did not increase. Activities reported at follow-up were more varied than at baseline, including some activities measured poorly by accelerometers (eg, biking and swimming). Participants suggested simplifying the website and incorporating other technologies.

**Conclusions:**

Good retention and increases in self-reported activity suggest a promising approach to delivering a physical activity intervention to Latina adolescents. Incorporating other technologies, such as smartphone apps, could make the intervention more engaging, acceptable, and effective.

## Introduction

The health benefits of physical activity for children and adolescents are extensive and include improved cardiovascular and metabolic health, decreased rates of obesity, and improved mental health [1]. To realize these health benefits, physical activity guidelines recommend that school-aged children engage in at least 60 min of moderate to vigorous physical activity (MVPA) on at least 5 days per week [2]. Rates of meeting guidelines in children in the United States, however, are quite low, particularly among adolescents. Although 42% of children aged 6 to 11 years meet guidelines based on objective measures, this drops sharply to only 8% in adolescents [2]. As health habits developed during adolescence predict health behavior and status later in adulthood [3-5], low rates of MVPA during this period could translate to insufficient MVPA for many years.

Although participation in MVPA is low during adolescence, it is especially low for girls, particularly racial/ethnic minority females. Compared with 17.9% of Mexican American boys, only 2.9% of adolescent Mexican American girls meet activity guidelines [6]. Paralleling this, Latina adults report less MVPA than non-Latino white and non-Latino black women [7] and are at higher risk of chronic diseases related to inactivity including overweight/obesity [8] and diabetes [9]. Developing effective interventions to increase MVPA in Latina adolescents is thus essential to promote health throughout the life course and reduce growing disparities.

Despite the low rates of activity and high risk of chronic disease in young Latinas, few MVPA interventions have targeted this group. Evaluations of past interventions showed that simply providing more information or more opportunities to be active was not effective in changing MVPA habits in Latino children [10,11], emphasizing a need for new theory-based and tailored interventions. Interventions grounded in psychosocial theories may be especially appropriate for increasing MVPA in youth, as self-efficacy and social support consistently emerge as the strongest correlates of MVPA in adolescents [12,13], and are consistently lower in adolescent girls than in boys [14,15]. Translating theory-based interventions into a Web-based format could be both appealing to adolescents and have potential for broad dissemination. Recent data showed that 92% of adolescents reported accessing the Internet daily, and 82% of Latino adolescents had access to a computer in their home [16].

We previously developed and tested a Web-based MVPA intervention for Latina adults (*Pasos Hacia La Salud*), which effectively increased MVPA over 6 months [17] and maintained increases 6 months later [18]. This intervention was adapted for Latinas and grounded in Social Cognitive Theory and the Transtheoretical Model, and could thus be an appropriate intervention approach for Latina adolescents. In this study, we modified this Web-based intervention based on formative research to ensure appropriateness for Latina adolescents and then tested the modified intervention in a 12-week single-arm demonstration trial. The aim of this study was to report the feasibility, acceptability, and potential efficacy of this theory-based, Web-delivered intervention for Latina adolescents.

## Methods

### Participants

The study sample comprised 21 Latina girls aged 12-18 years. Participants were eligible to enroll in the study if they (1) identified as Latina; (2) could read, write, and speak English fluently; (3) were physically inactive (≤90 min/week MVPA); and (4) had regular access to the Internet. Participants were ineligible for the study if they reported a health condition that would make unsupervised physical activity unsafe (according to the Physical Activity Readiness Questionnaire [19]). The study received human subjects approval from the University of California, San Diego’s Institutional Review Board. All participants gave written informed assent, and parents/guardians provided consent.

### Recruitment

The primary mode of recruiting participants was through health-focused community events, including health fairs, onsite school presentations, and church youth group meetings in San Diego. We also contacted adult Latinas who participated in the *Pasos Hacia La Salud* study to inquire if they had family or friends who could be eligible for the study. Other methods of recruitment included participant referrals and advertisements posted in churches, gyms, grocery stores, high schools, and parks.

### Protocol Overview

After a screening interview in person or via telephone to determine eligibility, participants came to an orientation session with their mother (or other primary caregiver) to learn about the study and complete the informed consent process. Participants were given an ActiGraph GT3X+ accelerometer (ActiGraph, Pensacola, FL), with instructions to wear it the following week. One week after the orientation visit, participants returned with the accelerometer and a completed packet of psychosocial questionnaires. Certified staff then administered the 7-day physical activity recall (PAR) interview to assess self-reported physical activity. Participants then received the individually tailored intervention, including a goal setting session and access to the personalized intervention website for 12 weeks. Check-in calls were made at 1 week and 1 month. Follow-up visits were completed approximately 12 weeks after study initiation.

### Tailored Intervention

The *Niñas Saludables* Web-based intervention was adapted from the *Pasos Hacia la Salud* intervention for adult Latinas. Semistructured interviews with 11 Latina girls, aged 12-19 years, were conducted to modify the content of the website. Similar to adult Latinas, the main theme across all age groups was finding time to exercise, followed by wanting support and not feeling motivated. As girls emphasized the importance of support, we included information on support throughout the website and challenged them to identify sources of support for that week’s goals. Girls also interacted with and commented on the *Pasos Hacia la Salud* website to guide intervention modification and make it more appealing for this age group. Main themes in the website feedback included needing more pictures throughout the website, particularly pictures that featured girls their age. Participants also reported wanting a more colorful template and reducing the amount of writing to provide a less cluttered look. The website was then modified to include more pictures of girls being active and a simplified homepage with highlighted shortcuts to goal setting and activity reporting. All tip sheets were redesigned to be more visual and reduce the amount of writing. We also included links to exercise videos for activities they expressed interest in, including Zumba and hip-hop dance. The website was then formatted to be mobile phone friendly, as many of the girls reported that they would only be accessing the website on their cell phones.

The first part of the intervention was a one-on-one goal setting session based on principles of motivational interviewing. A trained interventionist taught participants to set specific physical activity goals and performed guided problem-solving. Participants were given a pedometer to wear daily and were encouraged to track their steps and minutes of activity on a logging calendar on the website. All participants then received access to the *Niñas Saludables* study website for 12 weeks. The intervention was based on Social Cognitive Theory and the Transtheoretical Model and emphasized behavioral strategies for increasing activity levels (eg, goal setting, self-monitoring, and increasing social support). Intervention components included Internet-delivered activity manuals that were matched to participants’ current level of motivational readiness, computer-expert system tailored reports, activity tip sheets, and a guide of local activity resources. The tailored reports and activity tip sheets were both updated to reflect activities and common barriers that girls had reported in the formative interviews, such as dancing and how to resolve time conflicts.

**Figure 1:**
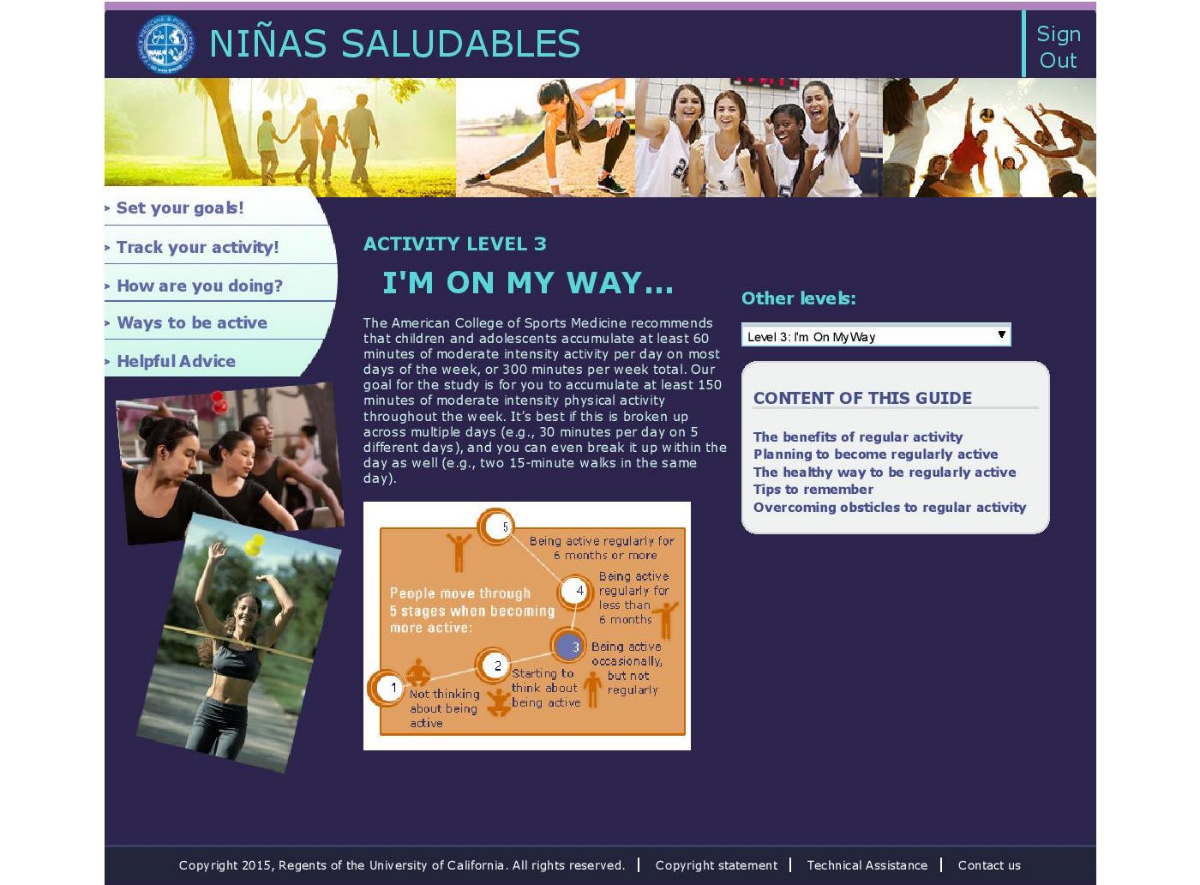
Screenshot of stage-matched moderate to vigorous physical activity information page.

**Figure 2:**
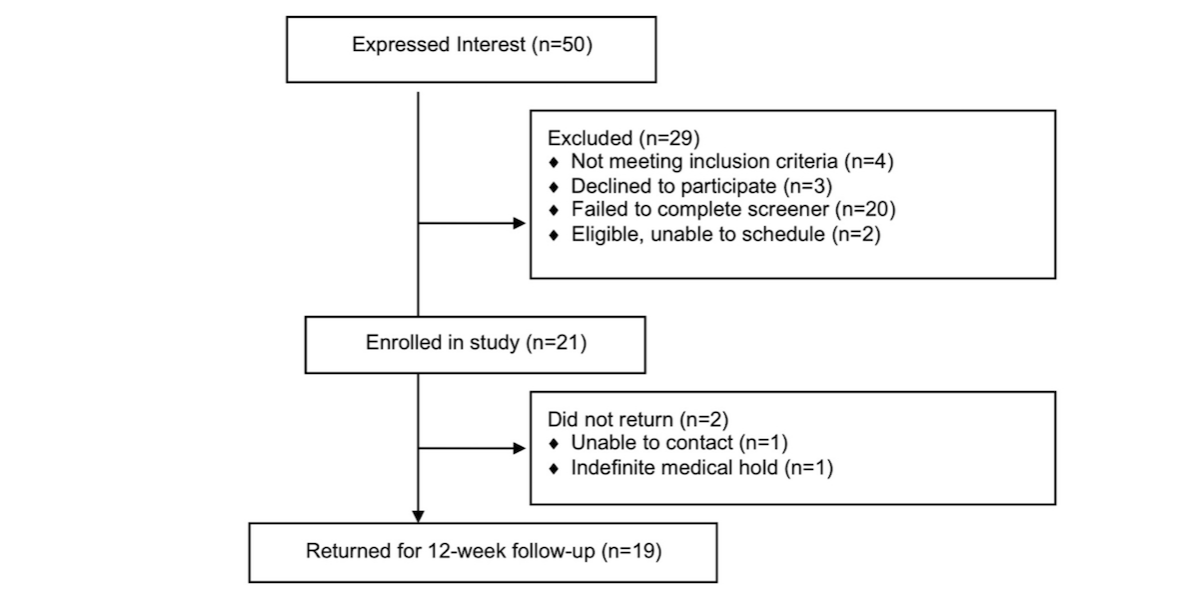
Recruitment and retention of participants.

Participants completed monthly questionnaires on the website (see the section Measures) that generated individually tailored content: (1) stage-matched webpages provided information about MVPA that was matched to the participant’s level of readiness, or stage of change, for becoming more physically active, according to the Transtheoretical Model and Social Cognitive Theory (see Figure 1), and (2) personalized computer expert system reports. The expert system draws from a bank of 330 messages addressing psychosocial and environmental factors influencing MVPA and automatically generates personalized reports on (1) their current stage of motivational readiness for physical activity; (2) increasing self-efficacy for physical activity; (3) cognitive and behavioral strategies associated with physical activity behavior change (processes of change); (4) how the participant’s answers compared with their prior responses (progress feedback); (5) how the participant’s responses compared with other adolescents who are physically active (normative feedback); and (6) self-monitoring of physical activity behavior (using online activity logging calendars). This expert system has been used in multiple intervention studies, and targeted theoretical constructs were shown to mediate changes in physical activity [20,21].

In addition, participants received tip sheets addressing barriers identified by Latina adolescents, such as support from family and friends, beating boredom, and finding time to exercise. New tip sheets were made available on a weekly basis during the first month, then biweekly during months 2 and 3. Per feedback from the formative interviews, participants were also sent email reminders when a new tip sheet was available. The local activity resource guide on the website included information and links to free and low-cost activity resources in the area, such as recreation center fitness schedules and mapped walking and hiking routes.

We also developed intervention materials for mothers to help them support their daughters. On a monthly basis, mothers were sent tip sheets on topics identified in formative interviews and literature to be important for supporting MVPA behavior change in their daughters, including positive support strategies, helping daughters choose activities, and tips for getting the whole family active. Tip sheets were available in English and Spanish.

### Measures

Adolescent’s weight, height, and resting blood pressure were collected at baseline. Parents provided basic demographic information (income, marital status, and family size) and filled out the Brief Acculturation Scale, which assesses language use across 4 life contexts [22]. All adolescent participants were fluent in English and were all relatively highly acculturated; thus, we did not assess acculturation in adolescents.

Psychosocial and environmental access variables were used for both evaluation and intervention-tailoring purposes, and all were assessed at baseline and post-test. The PACE+Adolescent Psychosocial measure assessed motivational stage for MVPA change (3 items), MVPA change strategies (15), self-efficacy (6), decisional balance (10), family influence and support (4), peer influence and support (6), enjoyment for activity (2), and environmental access (4). Items in each domain included questions that participants rated 1-5, with options differing by domain. For example, for self-efficacy, participants responded to questions such as, “do you feel you can do physical activity even when you feel sad or stressed?” (1: I’m sure I can’t, 5: I’m sure I can), or for family support, “during a typical week, how often has a member of your household done a physical activity or played sports with you?” (1: never, 5: every day). This measure has been shown to be reliable in an ethnically diverse sample of children as young as 11 years [23]. The Stages of Change for Physical Activity, Processes of Change, and Self-efficacy for Physical Activity were administered monthly on the website to generate stage-matched manuals and expert system reports. These 3 measures have been used extensively in physical activity research [24,25].

A consumer satisfaction questionnaire used in our past trials [18,26] was adapted specifically to assess the feasibility and acceptability of the current intervention.

### Physical Activity Measurement

Physical activity was measured at baseline and 12 weeks using the 7-day PAR, a semistructured interview to assess the frequency, duration, and intensity of MVPA, which has been validated against objective measures and shown to be sensitive to change over time [27-29]. At baseline and follow-up, this was preceded by a 10-min treadmill walk or walk outdoors to demonstrate what MVPA should feel like. Activity was also measured objectively using ActiGraph GT3X+accelerometers. Accelerometer wear was considered sufficient if participants wore it for ≥10 hours/day on ≥5 days. After processing accelerometer files using the Choi 2008 wear time validation algorithm [30], wear time was further visually examined to determine whether the accelerometer was in fact worn during short (<3 hours) and long (≥16 hours) periods of continuous wear. Once examined and unnecessary data categorized as nonwear, the data were scored using procedures identified by Treuth et al [31], which have been validated specifically for adolescent females.

### Data Analysis

Feasibility was determined by considering recruitment, retention, and adherence to the intervention at 12 weeks. For recruitment, we considered the number of people who needed to be contacted and screened to enroll 20 participants and the proportion of interested individuals who were deemed ineligible. The study was considered feasible if at least 80% of participants were retained, defined as attending the 12-week assessment. Adherence was determined by the percentage of monthly questionnaires that were completed and the percentage of materials participants reported reading. Acceptability was assessed by the satisfaction with the intervention at 12 weeks. The intervention was considered acceptable if at least 75% of participants indicated they were satisfied/very satisfied with the program.

The primary aim was to assess feasibility and acceptability; thus, we did not calculate power to detect efficacy. However, we examined changes in MVPA to explore potential efficacy. Unadjusted within-subject changes in MVPA were examined using *t* tests, and generalized linear models were used to assess changes in MVPA from baseline to 12 weeks (for both reported and objectively measured MVPA). Models use a likelihood-based approach to estimation and thus make use of all available data without directly imputing missing outcomes. All accelerometer data were adjusted for wear time. We tested the sensitivity of the findings to completers only and found no significant differences (*P*>.05 for sensitivity parameter). Our results present the intent-to-treat analyses with alpha level set at .05.

## Results

### Recruitment

A total of 50 individuals expressed interest in the study. The majority of these (n=39) came from community events, such as health fairs or school presentations. The next most successful recruitment strategy was contacting women who had recently completed the *Pasos Hacia la Salud* study (n=8). Passive recruitment, through advertisements posted in community locations, yielded only 2 calls. Another participant referred the final individual.

Of the 50 individuals who expressed interest, 30 were screened, 26 were deemed eligible, and 21 were enrolled in the study (see Figure 2). Participant yield was lowest from health fairs, where parents often signed up on behalf of their daughters, and we were unable to contact daughters to complete screenings. Enrollment was highest from presentations at schools and other sites where adolescents were directly targeted. Reasons for ineligibility included too much activity (n=2) and medical conditions (n=2).

### Baseline

Baseline characteristics are displayed in Table 1. The sample comprised 21 adolescent Latinas, aged 12-18 years (mean age=14.7, SD=2.1). Body mass index (BMI) varied greatly, ranging from underweight (BMI=17.4) to obese (BMI=33.5), with a mean BMI of 25.3. Families were generally low income, with 38% (8/21) reporting an annual family income below US $20,000. Overall, mothers had low acculturation levels, with over two-thirds reporting being the first generation in the United States, and the majority (76%, 16/21) speaking only Spanish or more Spanish than English.

Physical activity at baseline was relatively low, with a mean of 24.7 min/week of MVPA measured by the 7-day PAR (Table 1), and 24% (5/21) of girls reporting no activity at all. One outlier (4+ SDs above mean) was excluded from analysis. A large percentage of participants (28%, 6/21) had insufficient accelerometer wear time at baseline. Mean objectively measured MVPA at baseline was 24.8 (SD 38.3) in the entire sample and 21.1 (30.1) min/week among the subsample with sufficient wear time. Baseline stage of change was evenly split between contemplation (those thinking about becoming physically active) and preparation (those doing some activity, but not regularly). Of those reporting any activity at baseline, the majority (71%, 10/14) reported walking as their primary activity.

Girls reported relatively high levels of enjoyment for physical activity (mean=3.47; range 1-5), but low levels of family and friend support (2.06 and 1.84, respectively). Psychosocial variables were modestly correlated with baseline physical activity (see Table 2). The strongest correlates of baseline activity were self-efficacy (*r*=.34) and number of physically active friends (*r=*.32).

After 1 month, 81% (17/21) completed a check-in call, of which 59% (10/17) reported meeting their initial goals. The most commonly cited barrier for MVPA by far was time (70%, 12/17), followed by low motivation/energy (18%, 3/17), finding activities to do (6%, 1/17), and weather (6%, 1/17). None mentioned environmental barriers to activity. The most common source of support identified was their mothers (43% 6/14), followed by friends (29%, 4/14), other family members (21%, 3/14), and teachers (7%, 1/14).

### Twelve-Week Follow-Up

#### Feasibility

Of the 21 participants who began the study, 19 (90%) returned for the 12-week assessment. One was placed on an indefinite medical hold due to serious illness, and one could not be contacted again (Figure 2). This exceeded the predefined threshold of 80%, suggesting good feasibility. Adherence to the intervention, however, was moderate. Participants logged on to the website on a mean of 4.29 days (SD=3.24) and visited a mean of 32.2 different pages on the website (SD=15.9). Approximately two-thirds (63%, 40/63) of monthly questionnaires were completed. Only 42% (8/19) reported reading most/all of the individually tailored reports, and 42% (8/19) reported reading at least half of the additional emailed tip sheets.

**Table 1:** Baseline characteristics of study sample.

Baseline characteristic			Statistics
Study sample, N			21
Age, mean (SD)			14.7 (2.1)
Body mass index, mean (SD)			25.3 (4.2)
**Annual family income, n (%)**			
	<US $20,000		8 (38)
	US $20,000-$40,000		8 (38)
	>US $40,000		5 (24)
**Parent’s marital status, n (%)**			
	Married/living with partner		10 (47)
	Never married		4 (20)
	Separated/divorced		7 (33)
**Parent’s acculturation level**			
	**Generation status, n (%)**		
		First generation	14 (67)
		Second or third	7 (33)
	**Language, n (%)**		
		Mostly/only Spanish	16 (76)
		Spanish and English	3 (14)
		Mostly English	2 (10)
**Number of other children living in the home, n (%)**			
	0		0 (0)
	1		8 (38)
	2		5 (24)
	3+		8 (38)
**Minutes/week of MVPA^a^, mean (SD)**			
	7-day physical activity recall		24.7 (26.1)
	ActiGraph GT3X+ accelerometer		24.8 (38.3)
**Baseline stage of change, n (%)**			
	Contemplation		10 (47)
	Preparation		11 (53)
Self-efficacy (1-5), mean (SD)			2.82 (0.6)
Physical activity enjoyment (1-5), mean (SD)			3.47 (0.7)
Environmental access (1-5), mean (SD)			3.39 (0.7)
Family support (1-5), mean (SD)			2.06 (0.8)
Friend support (1-5), mean (SD)			1.84 (0.9)
**Number of physically active friends, n (%)**			
	0		0 (0)
	1		6 (28)
	2		6 (28)
	3+		9 (44)

^a^MVPA: moderate to vigorous physical activity.

**Table 2:** Predictors of baseline weekly minutes of activity (7-day physical activity recall).

Baseline psychosocial measures	*r*
Self-efficacy	.34
Family support	.28
Friend support	.29
Number of active friends	.32
Environmental access	.01
Physical activity enjoyment	−.01

#### Acceptability

A total of 18 participants completed final consumer satisfaction surveys. Overall satisfaction was moderate, with 72% (13/18) of participants saying they were satisfied/very satisfied with the program, 28% (5/18) indicating they were dissatisfied, and none saying they were very dissatisfied. Similarly, 72% (13/18) said they were likely/very likely to recommend the program to family or friends. This was just below the predefined threshold of 75%. Participants rated the most useful features of the website (in order) to be (1) the goal setting calendar, (2) the activity logging calendar, and (3) the information on local activity resources. The message board and Ask the Expert forum were rated the least helpful.

The most common complaint about the program was having to log in to use the website, particularly when accessing the website through a smartphone. Participants expressed enthusiasm for expanding to other media channels, particularly a smartphone app and/or texting, and making the website more “youthful” and decreasing the amount of writing. Another common theme was to make the program more social; suggestions included enrolling friends together, incorporating teams or clubs, offering it at a school, and expanding the program to all girls, not just Latinas.

#### Physical Activity

**Figure 3:**
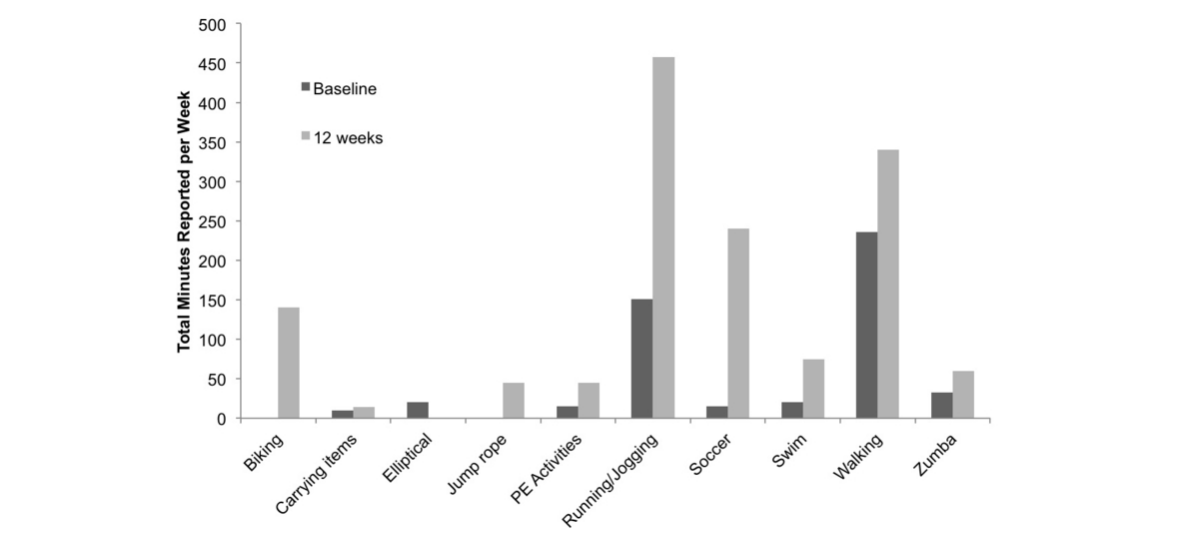
Total weekly minutes reported in different activities at baseline and follow-up. PE: physical education.

Measured by the 7-day PAR, participants increased weekly minutes of MVPA from a mean 24.7 (SD 26.1) at baseline (range: 0-85) to 79.4 (SD 46.8) at follow-up (range: 14-177), with a mean increase of 58.8 (SD 46.3) min/person (*P*<.001; range: 4-155). Likelihood-based estimates showed significant increases baseline to 12 weeks (*P*<.001). All participants who completed follow-up visits reported doing some physical activity, ranging from 14 to 177 min/week. Activity types reported were more varied than at baseline and included running, walking, soccer, Zumba, swimming, and cycling. Accelerometer-measured MVPA decreased from 24.8 min/week at baseline to 10.4 (SD 30.2) at 12 weeks. Some girls who reported high activity at follow-up participated in activities in which the accelerometer could not be worn (eg, swimming or contact sports) or that were not well measured by the accelerometer (eg, cycling). Figure 3 shows total weekly minutes reported across the sample for different types of activities at baseline and 12-week follow-up.

## Discussion

### Principal Findings

These data suggest good feasibility of the MVPA intervention for Latina adolescents, as demonstrated by the study recruitment and retention rates. Moreover, self-reported MVPA results suggest the potential efficacy of the *Niñas Saludables* intervention in increasing MVPA among Latina adolescents. These results are consistent with previous studies that have successfully used adaptations of this intervention to promote MVPA among other populations, including adult Latina women [32-34].

Although the theory-based intervention was modified based on feedback from the target population, qualitative interviews and consumer satisfaction questionnaires conducted on intervention completion showed that *Niñas Saludables* was only somewhat acceptable for Latina adolescents, and additional modifications may be necessary to increase acceptability. A recurring theme from postintervention interviews was the need to further tailor the reports and stage-matched manuals to be more age-specific and concise. Girls expressed a preference for shorter reports more often, as opposed to longer reports once per month. This was also captured by the low adherence rates, with participants reading a relatively small amount of the information provided in response to their monthly assessments. These findings are consistent with the literature: overall, Internet-based interventions for the promotion of physical activity have been found to be effective [35], yet adherence remains an obstacle that may account for small effect sizes [36]. Previous research has found that harnessing the persuasive capability of technology (using tools such as tailoring, rewards, and competition) improves adherence to Web-based interventions.

Web-based interventions will need to further capitalize on the use of technological tools, which have become increasingly popular with the rise of mobile health (mHealth) [37]. For example, use of multimedia elements like videos and images has been shown to increase understanding and improve performance in a variety of behaviors and populations [38-40], and could address the common complaint in this study of having too much writing on the website. *Niñas Saludables* participants endorsed the possibility of using newer technologies, including smartphone apps and texting, which could circumvent the need to log in to a website. Interactive features such as goal setting and activity logging were identified as the most useful features, along with the personalized reports and goal-setting session. Given our increasing capability to incorporate technology and interactive features in promoting physical activity, future research should aim to include more of these features in interventions to appeal to this specific population, while maintaining theory-based strategies to promote behavior change. Future qualitative research may be necessary to understand how to incorporate these elements and how to develop effective physical activity interventions that address the needs of Latina adolescents, and to further investigate the role of environmental access and enjoyment, which were surprisingly unrelated to MVPA in this study. To gain greater insight into the feasibility of technology-based MVPA interventions among young Latinas, consumer satisfaction measures in future studies can also include an evaluation of social acceptability as described in the study by Poder et al [41].

Results from the 7-day PAR showed that changes in activity at 12 weeks were seen not just in quantity but also in type. Increases in self-reported MVPA in our study were comparable to those found in *Pasos Hacia la Salud* at 6 months (ie, mean increases in min/week MVPA 58.8 vs 50, respectively); however, changes in accelerometer-measured MVPA differed substantially between the 2 studies. A potential explanation for the decrease in objectively measured MVPA in *Niñas Saludables* is that the broad range of activity types that participants reported engaging in at 12 weeks may not be accurately measured using accelerometers (eg, swimming, cycling). Although activity at baseline was mostly restricted to walking, activities reported at follow-up were more varied. This could be in response to intervention materials that specifically encouraged trying new activities, including tip sheets for keeping activity interesting, tip sheets for mothers to help daughters find new activities, and the activity resource guide, which provided information on sports and activity classes in the community. This could be an important feature of interventions, as previous literature suggests individuals who enjoy activity are more likely to increase and maintain their MVPA. These findings are contrary to the results from previous studies among adult Latina women, who have mostly increased walking as a result of similar interventions [33,42], although walking was still a popular activity in our study. Our findings are thus encouraging and merit further research to understand the different results obtained among adolescent Latinas compared with adult Latinas, and whether engagement in activities other than walking may result in increased enjoyment and maintenance of MVPA. Our findings also differ from previous studies in that Latina adolescents did not endorse incorporation of Latino culture into intervention components and expressed preference for a program their non-Latina friends could also participate in. An intervention targeted to all adolescent girls could be more generalizable and prevent Latinas from feeling singled out.

Our findings also revealed challenges associated with the measurement of physical activity among Latina adolescents. Compliance with accelerometer wear was low compared with our previous studies [17]. Accelerometers may also have underestimated activity, as participants could not wear accelerometers during certain activities such as swimming or contact sports, and activities such as biking may have been inadequately captured by accelerometers. Given the high proportion of participants who reported engaging in these types of activities, accelerometry provided an incomplete measure of MVPA. Moreover, there are a wide range of suggested MVPA accelerometer cut points for adolescents, and there is no consensus on which is most appropriate [43-45]. Although the cut points used here were validated specifically for adolescent girls, the validation study found that cycling and step aerobics had poor calibration with accelerometers and found a wide range of metabolic equivalents in individuals for each level of accelerometer counts. These difficulties emphasize the importance of continuing to use validated self-report measures, both to measure quantity and type of activity, and to improve tools of objective measurement of MVPA among this and similar populations. Girls in our study also reported a good amount of activity in 10-min bouts, which, if it were slightly less than 10 min, would not have been counted by accelerometers. Few girls participated in the treadmill walk at follow-up, which may have also increased reporting of activities that were not moderate intensity. Social desirability could have also influenced self-reported physical activity. Options for objective assessment of physical activity are expanding and include integration of geographic positioning system, accelerometer, and geographic information system data to reveal where people are active; use of heart rate monitors along with accelerometers; and devices such as ActivPAL to collect complementary data on sedentary behavior [46]. However, all these options require wearing additional devices, which increases participant burden.

### Strengths and Limitations

Limitations of this study included the small sample size and single-arm design, which were appropriate for the current feasibility study but prevented formal efficacy analyses or more detailed explorations of data, including potential mediators and moderators. Given the preliminary nature and small sample size of this pilot study, results cannot be generalized to a broader population of Latina adolescents or to locations outside the research setting. Results need to be replicated among larger samples in a randomized trial with a control group to determine whether this intervention is efficacious in helping Latina adolescents increase their MVPA. Strengths of this study included the novel, high-risk population; Web-based intervention channel; formative research; and individually tailored, theory-based intervention content.

### Conclusions

The findings detailed here are promising, particularly given the paucity of research regarding the promotion of MVPA among Latina girls and adolescents and the importance of MVPA to lifelong health. These data suggest that delivering an individually tailored Web-based physical activity intervention to Latina adolescents is feasible and has potential to be efficacious, particularly when the intervention is made more acceptable by incorporating more mHealth strategies and technologies that are becoming increasingly available on a broad scale in this and other populations.

## Abbreviations

BMI: body mass index
mHealth: mobile health
MVPA: moderate to vigorous physical activity
PAR: physical activity recall
